# A comparison between the effectiveness of a gamified approach with the conventional approach in point-of-care ultrasonographic training

**DOI:** 10.1186/s12909-020-02173-7

**Published:** 2020-08-12

**Authors:** Aaron Kuo Huo Lai, Abdul Muhaimin bin Noor Azhar, Aidawati binti Bustam, Xun Ting Tiong, Hiang Chuan Chan, Rashidi bin Ahmad, Keng Sheng Chew

**Affiliations:** 1 Emergency and Trauma Department, Sarawak General Hospital, 93586 Kuching, Sarawak Malaysia; 2 Faculty of Medicine, University of Malaya, Jalan Universiti, Wilayah Persekutuan Kuala Lumpur, 50603 Kuala Lumpur, Malaysia; 3 Clinical Research Centre, Sarawak General Hospital, 93586 Kuching, Sarawak Malaysia; 4 Faculty of Medicine and Health Sciences, Universiti Malaysia Sarawak, Kota Samarahan, 94300 Kuching, Sarawak Malaysia

**Keywords:** Gamification, Emergency medicine, Ultrasound training

## Abstract

**Background:**

Although gamification increases user engagement, its effectiveness in point-of-care ultrasonographic training has yet to be fully established. This study was conducted with the primary outcome of evaluating its effectiveness in point-of-care ultrasonographic training as compared to conventional approach.

**Methods:**

Participants consisting of junior doctors were randomized into either the (1) gamified or the (2) conventional educational approach for ultrasonographic training.

**Results:**

A total of 31 junior doctors participated in this study (16 participants in gamified arm, 15 in the conventional arm after one participant from the conventional arm dropped out due to work commitment). Two-way mixed ANOVA test showed that there was no statistically significant interaction between the types of educational approach and time of testing (pre-test, post-test, 2 months post-training) for both theoretical knowledge score and practical skills score, with F(2, 58) = 39.6, *p* < 0.001, partial η^2^ = 0.4 and F(2, 58) = 3.06, *p* = 0.06, partial η^2^ = 0.095, respectively. For theoretical knowledge score, pairwise comparisons showed that the mean 2 months post-training scores (20.28 +/− 0.70, 95% CI 18.87–21.69) and mean post-test scores (20.27 +/− 0.65, 95% CI 18.94–21.60) were better than the pre-test scores (12.99 +/− 0.50, 95% CI 11.97–14.00) with *p*-values < 0.001 for both comparisons respectively. Similarly, for practical skill score, pairwise comparisons showed that the mean 2 months post-training scores (20.28 +/− 0.70, 95% CI 18.87–21.69) and mean post-test scores (20.27 +/− 0.65, 95% CI 18.94–21.60) were also better than the pre-test scores (12.99 +/− 0.50, 95% CI 11.97–14.00) with *p*-values < 0.001 for both comparisons respectively. Participants in the gamification arm generally perceived the various game elements and game mechanics as useful in contributing and motivating them to learn ultrasonography.

**Conclusions:**

Gamification approach could be an effective alternative to conventional approach in point-of-care ultrasonographic training.

## Background

Point-of-care ultrasound (POCUS) is a vital diagnostic and therapeutic intervention for acute patient care [1]. It is a goal-directed, focused and limited ultrasonographic examination performed at the patient’s bedside to answer specific clinical questions within a reasonable amount of time [2]. The Rapid Ultrasound for Shock and Hypotension (RUSH) protocol, for example, is a 3-step POCUS approach aimed to identify causes of a patient with undifferentiated shock [3].

Due to its importance and practicality, POCUS training has been incorporated as a core competency in emergency and critical care [1]. It is effective when conducted in a small group setting using video clips and hands-on scanning sessions [4]. Even a brief, one-day session has been shown to be effective [5]. The skills acquisition from such trainings are conventionally assessed using manikins, simulated patients or computer simulators [6].

Gamification is defined as “the use of game design elements in a non-game context” [7]. By embedding the element of fun through the use of game components such as the points, badges and leader boards [8], gamification has been shown to improve user engagement, focus, motivation as well as productivity and knowledge retention [9, 10]. A number of gamified POCUS trainings have been described, including the Sound Games [11], the SonoGames [12] and the “Sono-Witcher Wild Hunt” [13]. Yet, to the best of our knowledge, there is a paucity of literature describing the comparison between a gamified approach and the conventional approach in POCUS training. This study sought to address this gap. The primary objective of this study was to compare the effectiveness of a competition-based gamified POCUS training (known as the “Competition-based Rapid Ultrasound in Shock and Hypotension or ‘CRUSH’ Games) with that of the conventional approach for POCUS training. The secondary outcomes of this study were 1) to assess the overall knowledge and skill retention of 2 months post-training and 2) to assess the participants’ perception of the gamification experience in POCUS training.

## Methods

### Study design and setting

This study was a randomized trial conducted in the Emergency and Trauma Department (ETD) of Sarawak General Hospital (SGH), Malaysia to compare the gamification approach versus the conventional approach for POCUS training using the RUSH protocol. An assessment of the participants’ perception towards gamification approach in POCUS training was also included. This study was approved by the Medical Research and Ethics Committee (MREC) Malaysia and was registered with the National Medical Research Register (NMRR-18-444-40,348). As it was conducted in a training workshop setting, the number of participants was limited by resource availability. Hence, a convenient sampling of 32 participants (16 participants in each arm) were recruited.

### Study population

The study population comprised of junior doctors working in (1) the ETD SGH, (2) Sarawak Heart Center and (3) the Internal Medicine Department of SGH. To recruit the participants, invitations were first sent out to the heads of departments of ETD SGH, Sarawak Heart Center and the Internal Medicine Department of SGH. The heads of departments would be given the onus to recommend participants for this workshop. Fourteen participants were recruited from the Internal Medicine Department of SGH, 13 participants came from the ETD SGH and another 5 participants were from the Sarawak Heart Center.

We defined a ‘junior doctor’ as a doctor with 2 to 4 years’ experience in clinical service. The reason for selecting doctors with 2 to 4 years of clinical experience was due to the fact that doctors with this amount of experience would have completed their compulsory two-year internship program in Malaysia and at the same time they would likely have developed sufficient clinical exposure to be able to utilize POCUS findings for clinical decision making.

Any junior doctor who had participated before in any formal POCUS training was excluded. Informed consent was obtained from all participants before commencing this study. All participants joined this free POCUS training on a voluntary basis without any payment or monetary compensation.

### Materials

The topics for this POCUS training workshop were based on the requirements of the original RUSH protocol adopted from the World Interactive Network Focused on Critical Ultrasound (WINFOCUS) Malaysia course as well as some adaptation from the emergency ultrasound training from a post-graduate emergency medicine training program in Malaysia (i.e., the Universiti Malaya Emergency Medicine postgraduate curriculum). All materials were internally validated via a modified Delphi technique to attain consensus by a panel of experts in emergency medicine. Modified Delphi technique is a structured iterative technique to obtain consensus from experts through rounds of email, online, face-to-face communication until consensus is reached [14]. The experts were emergency physicians in Malaysia, two of which were actively involved in giving POCUS training.

### Study procedure

This study was divided into two stages. i.e., (1) to identify learning materials and development of assessment questions using the modified Delphi method and (2) to conduct recruitment, randomization and implementation of two different educational approaches in a POCUS training. In the first stage, the discussions were carried out with a panel of three experts in three rounds. Most of the discussions were carried out via e-mail and online group dialogues as the experts were based in different locations in Malaysia. The first round of discussions focused on identifying the main objectives and the probable topics of the workshop. In the second round of discussion, this compiled list of probable topics was distributed by email to the experts for review and to reach a consensus on the suitability of the topics. In the final round of discussions, the shortlisted topics were divided and assigned to the specific panel of experts for teaching as well as for preparation for the assessment questions.

This assessment consisted of two sections: (1) 30 one-best answer (OBA) type of multiple-choice questions (for theory assessment), and (2) one objective structured clinical examination (OSCE) case scenario (for practical assessment). The OSCE case scenario was constructed in the form of a commonly seen clinical case such as “a man who was brought to the emergency department complaining of severe abdominal pain with unstable vital signs”. A volunteer would be trained to play the role of the simulated patient. The participants would then be instructed to demonstrate the technique of performing POCUS on the simulated patient. After demonstrating the technique, the assessor would display a series of ultrasonographic images on an electronic device and asked the participants to interpret these images as if these images were the findings of the simulated patient. All questions were then vetted, revised, finalized and agreed upon by the experts.

In the second stage of study, participant recruitment and randomization were conducted. Thirty-two participants were randomized to either the gamification group (known as the “CRUSH” group) or the conventional group. Each participant was first assigned a number. A free online random number generator (https://www.randomizer.org/) was then used to generate 2 sets of unique numbers. One set of numbers for the CRUSH group, and another set for the conventional group. The participants were then assigned to the different groups based on the numbers given to them earlier on.

On the first day of the course, all participants (regardless of which groups they were assigned to) were required to complete a pre-test knowledge (30 one-best answer (OBA) type of multiple-choice questions) and practical skills assessment test. The practical skills assessment was conducted in the style of OSCE case scenario, conducted using a simulated patient and was assessed by three independent emergency physicians who were blinded to the participants’ study arms.

**Fig. 1:**
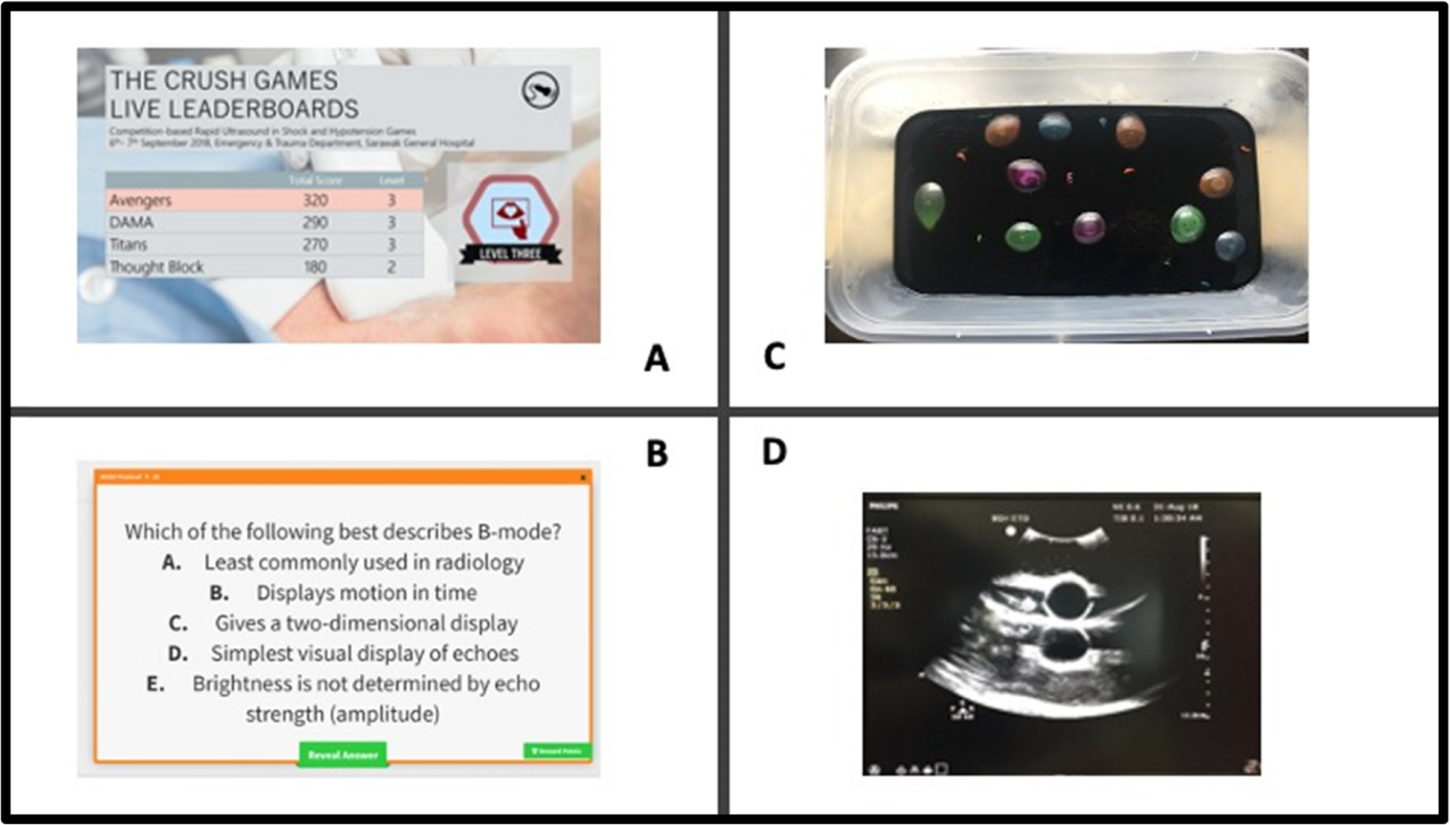
Examples of some of the game mechanics used in the CRUSH Games. **a**. The real-time live leader boards displayed during the course reflecting each team’s progression, level and scoring in the gamification arm **b**. A sample quiz question from the FlipQuiz™ platform. **c**. Ultrasound Minefield: Water balloons in a half-filled gelatin container (left image) and **d**. its appearance on ultrasound (right image)

All participants then attended the classroom lectures on topics related to POCUS, interspersed with knowledge assessment activities. For participants in the conventional arm, this assessment was conducted using written-type quizzes. Whereas, for participants in the CRUSH arm, this assessment was conducted in the format of a team-based competition-like live quizzes with different level of difficulties. Points (or known as e**X**perience **P**oints, XP) were allocated for each correct answer and the score was tabulated on a live leader board (Fig. 1a). Each team began at level one with zero XP and they would require sufficient XP to progress to the next level. XP points could be gained by answering the live quizzes interspersed between lecture modules. Virtual badges were rewarded based on progression. If team was not able to answer the question correctly, another team was given a chance to answer but this team could only attain half the value of XPs even if they answered it correctly. The quiz was presented using a Jeopardy-style game show format using FlipQuiz™ technology (Fig. 1b) whereby teams are able to pick the level of difficulty of questions with different points allocated. Feedback on the correct answers was given immediately to all teams after the stipulated time to answer the question was over. There was no negative marking in all quizzes.

On the second day, a demonstration session was first given to all participants (regardless of which groups they were assigned to) on the introduction to ultrasound machine, the probes and image acquisition. This is then followed by hands-on training sessions, conducted using simulated patients. For participants in the conventional group, these hands-on sessions were conducted in the form of the individual skill demonstration and practice sessions. No points or scores were awarded to the participants in the arm for these formative activities. For participants in the CRUSH arm, these hands-on skill trainings were conducted in the form of games, i.e., ultrasound minefield, ultrasound pong and ultrasound game. The psychomotor skill objectives covered in the hands-on practical sessions and the corresponding games for participants in the CRUSH arm are given in Table 1. At the end of the course, the team with the highest XPs score would win a reward. The XPs points collected, however, would not be carried into the calculation of the pre-test or post-test.

**Table 1:**
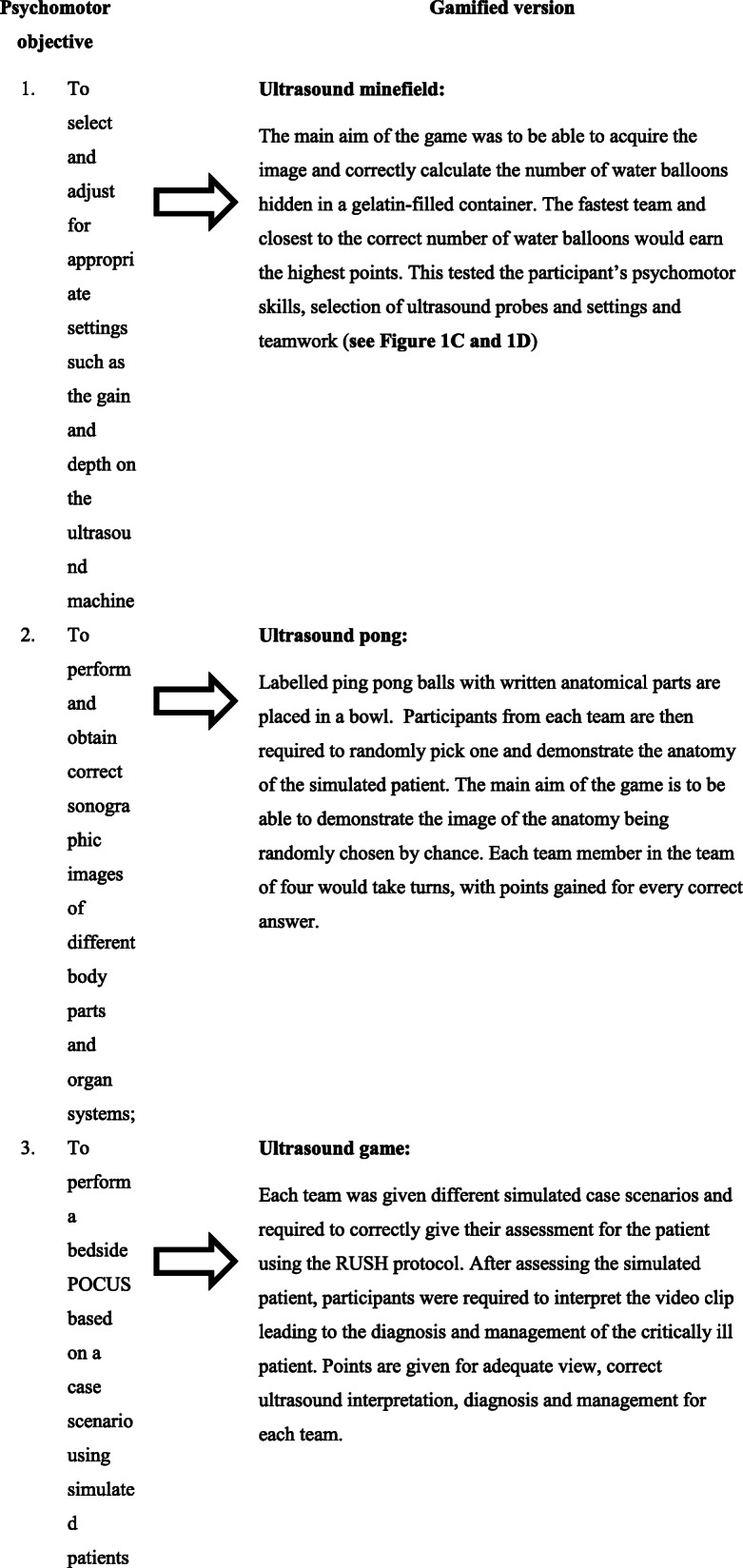
The psychomotor skill objectives in hands-on practical sessions and the corresponding gamified version

**Fig. 2:**
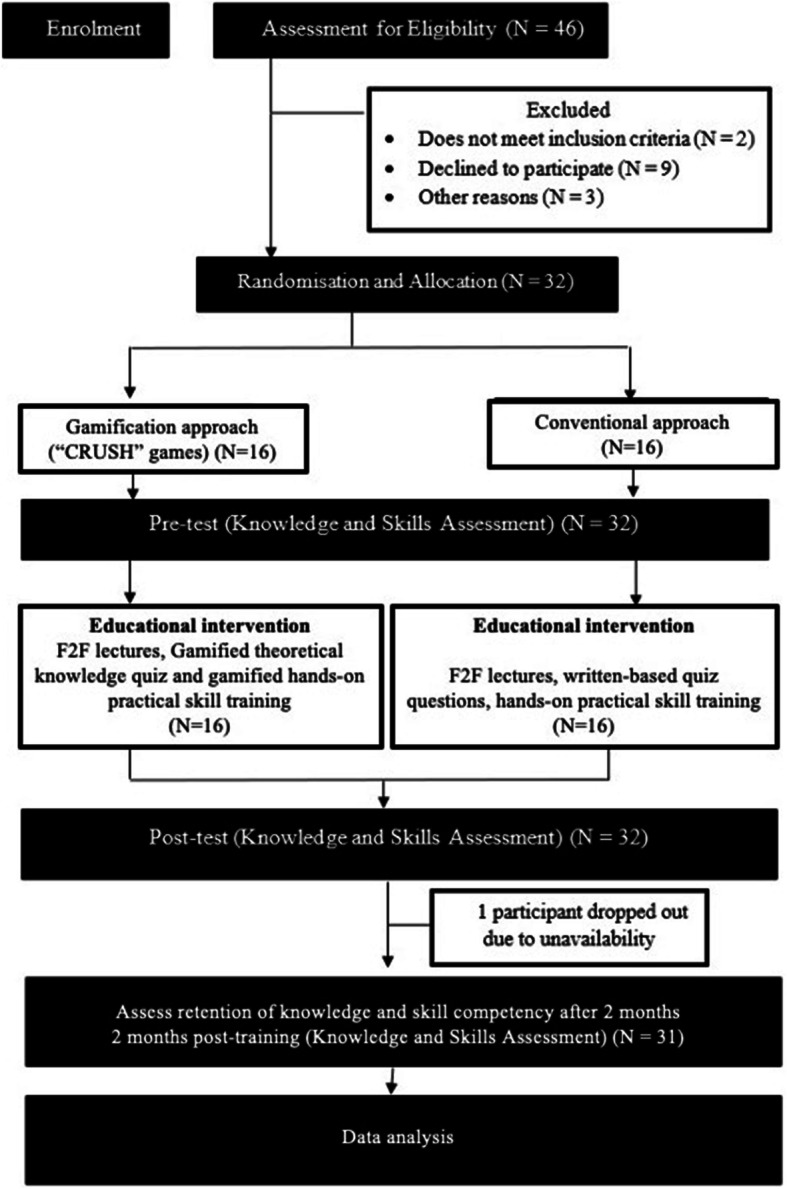
Flowchart of the overall process of data collection

After completing these hands-on sessions, a post-test (similar to the formats in pre-test, i.e., OBA questions for theory assessment and one OSCE case scenario, conducted using simulated patient, for practical skill assessment) was conducted. The overall flowchart of the 2-day POCUS workshop is given in Fig. 2 and the detailed contents and schedule of the workshop is given in Table 2.

**Table 2:** Two-day schedule for POCUS training with the RUSH protocol for both CRUSH and conventional groups

**No**	**Day 1: Lecture Modules**	**Duration**
**1**	Pre-test	1 h
**2**	Lecture: Introduction to ultrasound physics and knobology	30 min
**3**	Lecture: Introduction to the RUSH Protocol	30 min
**4**	Lecture: Abdominal scan/FAST	30 min
	**CRUSH Group: Round 1 Team-based competition quiz** **Conventional Group: Written-based quiz 1**	**15 min**
**5**	Lecture: Lung ultrasound	30 min
**6**	Lecture: Cardiac ultrasound	30 min
	**CRUSH Group: Round 2 Team-based competition-based quiz** **Conventional Group: Written-based quiz 2**	**15 min**
**7**	Lecture: Vascular ultrasound (Inferior vena cava, Aorta, 2-point compression test)	30 min
**8**	Lecture: Putting it all together	30 min
	**CRUSH Group: Round 3 Team-based competition-based quiz** **Conventional Group: Written-based question quiz 3**	**15 min**
**Station**	**Day 2: Hands-on Training**	**Duration**
**1**	Demonstration session: Introduction to machine, probes and image acquisition	30 min
	**CRUSH Group: Team-based competition ultrasound game Round 1: Ultrasound Minefield** **Conventional Group: Individual Skills Training: Acquiring image of water balloons**	4 mins per team 1 min per individual
**2**	2A: Cardiac ultrasound in RUSH protocol	40 min
	2B: Lung ultrasound in RUSH protocol	40 min
	2C: Abdomen ultrasound/FAST in RUSH protocol	40 min
	2D: Vascular ultrasound in RUSH protocol	40 min
	**CRUSH Group: Team-based competition-based ultrasound game Round 2: Ultrasound Pong** **Conventional Group: Individual Skill Training: Anatomy**	**5 mins per team** **1 min per individual**
**3**	Simulated Case-based Scenarios of Undifferentiated Shock: Application of RUSH protocol. **CRUSH Group: Team competition-based ultrasound game Round 3 (10 min per team)** **Conventional Group: Case-based classroom discussion**	**10 mins per team**
	3A: Case 1 - Cardiogenic Shock	10 mins
	3B: Case 2 – Obstructive Shock	10 mins
	3D: Case 3 – Hypovolemic Shock	10 mins
	3E: Case 4 – Distributive Shock	10 mins
	Post-test	1 h

*FAST* Focused assessment with sonography for trauma

To assess the participants’ retention of knowledge and skills, a similar theory and practical assessment was repeated 2 months after completion of the course. We chose a time gap of 2 months based on a previous study which shows that knowledge retention after an educational intervention was approximately 55 days or less [15]. The maximum score that could be obtained for all theory assessment and practical skill assessment were 30 marks and 25 marks respectively.

Two-way mixed ANOVA with one between-group factor (type of educational approach, i.e., conventional vs CRUSH) and one within-group factor (time of assessment, i.e., pre, post- and 2 months post-training) was used in this study. The data was approximately normally distributed based on visual inspection of the Q-Q plot and there were no outliers, as assessed by examining the studentized residuals for values greater than ±3. There was homogeneity of variances as demonstrated by Levene’s test of homogeneity of variances with *p* > 0.05 as well as homogeneity of co-variances as demonstrated by Box’s test of equality of covariance matrices (*p* = 0.90). The assumption of sphericity for the two-way interaction was met as demonstrated by the Mauchly’s test of sphericity with χ^2^(2) = 0.713, *p* = 0.700.

In addition, participants from the CRUSH arm also completed a gamification experience survey (adapted from Lobo et al., 2017) [4] aimed to assess the participant’s perception of the different components of gamification using a Likert scale. All the quantitative data was analyzed using IBM Statistical Package for the Social Sciences (SPSS) v23 for Windows.

## Results

A total of 32 junior doctors participated in this study with 16 participants randomized to each arm. One participant from the conventional was subsequently dropped out from the study (and analysis) during the 2 months post-training assessments due to work commitment. The mean age of our participants was 27 +/− 1.5 years old in both groups. In terms of gender, there were 5 (31.2%) male and 11 (68.8%) female participants in the conventional group. Similarly, there were 6 (37.5%) male and 10 (62.5%) female participants in the CRUSH group.

The two-way mixed ANOVA used to compare the mean differences of the repeated measures of practical skills (pre-, post- and 2 months post-training) between the conventional and CRUSH groups, showed that there was no statistically significant interaction between the types of educational approaches (conventional and CRUSH) and time (pre-test, post-test, 2 months post-training) on the practical skills score, F(2, 58) = 3.06, *p* = 0.06, partial η^2^ = 0.095. The main effect of time showed a statistically significant difference in mean practical skills score at different time measures with F(2, 58) = 39.6, *p* < 0.001, partial η^2^ = 0.421. Pairwise comparisons showed that mean 2 months post-training scores (20.35 +/− 0.61, 95% CI 19.10–21.60) and mean post-test scores (18.33 +/− 0.53, 95% CI 17.25–19.41) were better than the pre-test scores (14.55 +/− 0.89, 95% CI 12.73–16.37) with *p*-values < 0.001 for both comparisons. However, there was no statistically significant difference between the 2 months post-training scores and the post-test scores, with *p* = 0.07. The main effect of types of educational approaches also showed that there was no statistically significant difference in mean performance scores irrespective of time measures between these 2 types of educational approach with F(1, 29) = 2.38, *p* = 0.134, partial η^2^ = 0.08.

Similarly, two-way mixed ANOVA conducted to compare the mean differences of the repeated measures of theoretical knowledge (pre-, post = and post 2 month) between the conventional and CRUSH groups showed that there was no statistically significant interaction between the types of educational approach (conventional and CRUSH) and time (pre-test, post-test, post 2 months post-training) on theoretical knowledge scores, F(2, 58) = 3.06, *p* = 0.06, partial η^2^ = 0.095. The main effect of time showed a statistically significant difference in mean theoretical knowledge scores at different time measures with F(2, 58) = 39.6, *p* < 0.001, partial η^2^ = 0.421. Pairwise comparisons showed that mean 2 months post-training scores (20.28 +/− 0.70, 95% CI 18.87–21.69) and mean post-test scores (20.27 +/− 0.65, 95% CI 18.94–21.60) were better than the pre-test scores (12.99 +/− 0.50, 95% CI 11.97–14.00) with *p*-values < 0.001 for both comparisons respectively. However, there was no statistically significant difference between 2 months post-training scores and the post-tests scores, with *p* = 1.00. The main effect of types of educational approaches also showed that there was no statistically significant difference in mean theoretical knowledge score irrespective of time measures between these 2 types of educational intervention with F(1, 29) = 0.75, *p* = 0.40, partial η^2^ = 0.02. There was homogeneity of variances as demonstrated by Levene’s test of homogeneity of variances with *p* > 0.05 as well as homogeneity of co-variances as demonstrated by Box’s test of equality of covariance matrices (*p* = 0.90). The assumption of sphericity for the two-way interaction was met as demonstrated by the Mauchly’s test of sphericity with χ^2^(2) = 4.88, *p* = 0.09.

A subgroup analysis of the CRUSH participants’ perception towards the gamification experience survey was also performed using a Likert scale of 1 to 5. This subgroup analysis was divided into three components related to gamification, i.e., “engagement”, “perceived knowledge and learning benefit” and “game elements and mechanics”. Overall, the participants demonstrated positive perceptions to the various aspects of gamifications like better engagement and motivation, increased in self-perceived knowledge and learning benefits and enjoyable gaming elements and mechanics (teamwork, competition, points, badges, leader board, immediate feedback, rewards) introduced throughout the course. One particular game which was more favorited by the participants compared to the other types of games was the “ultrasound game”. All the participants in the CRUSH arm (*n* = 16, 100%) concordantly agreed that the hands-on ultrasound games were enjoyable during the course. The detailed responses of the participants gamification experience are described in Table 3.

**Table 3:** Participants responses to gamification experience survey

No	Item	Mean	Likert Scale Response (N, Percentage – (%))				
			Strongly Disagree	Disagree	Neutral	Agree	Strongly Agree
**1**	I feel motivated when participating in this course using the gamification approach	4.88	0	0	0	2 (12.5)	14 (87.5)
**2**	I feel that time passed by quickly during this course	4.88	0	0	0	2 (12.5)	14 (87.5)
**3**	Overall, I am satisfied with the overall content of delivery of the course	4.88	0	0	0	2 (12.5)	14 (87.5)
**4**	I believe that my knowledge of ultrasound improved over the course	4.94	0	0	0	1 (6.3)	15 (93.8)
**5**	I find that my ability to acquire images improved over the course	4.88	0	0	0	4 (25)	12 (75)
**6**	I am more confident in interpreting ultrasound images	4.62	0	0	0	6 (37.5)	10 (62.5)
**7**	I believe I can make clinical decisions better with the use of ultrasound after the course	4.75	0	0	0	4 (25)	12 (75)
**8**	I believe that the friendly competition helped me to retain more information from this 2-day course.	4.94	0	0	0	1 (6.3)	15 (93.8)
**9**	I enjoyed working with a teammate throughout the course	4.75	0	0	0	4 (25)	12 (75)
**10**	Group competition and workshop helped me to get to know my colleagues better	4.88	0	0	0	2 (12.5)	14 (87.5)
**11**	I am satisfied with the team seating arrangement of the course	4.69	0	0	0	5 (31.3)	11 (68.8)
**12**	The points system motivated me throughout the course	4.69	0	0	0	5 (31.3)	11 (68.8)
**13**	The leader board system motivated me throughout the course	4.69	0	0	0	5 (31.3)	11 (68.8)
**14**	A sense of competition motivated me during the course	4.88	0	0	0	2 (12.5)	14 (87.5)
**15**	I believe that immediate feedback from the quiz was helpful	4.81	0	0	0	3 (18.8)	13 (81.3)
**16**	The hands-on ultrasound games were enjoyable	5.0	0	0	0	0	16 (100)
**17**	The presence of rewards^a^ motivated me through the course	4.56	0	0	0	7 (43.8)	9 (56.3)

Items 1 to 3 represents perception of the engagement component.

Items 4 to 8 represents perception of perceived knowledge and learning benefit in the gamification approach.

Items 9 to 17 represents perception of game elements and game mechanics.

^a^The rewards in our case were the small dry food hamper given to the top three teams

## Discussion

The results from this study suggest that gamification approach in POCUS training may be as effective as conventional approach. This is evidenced by a statistically significant improvement in both theoretical knowledge and practical skill components from pre- to post-as well as 2 months post-training in both gamification arm and the conventional arm (besides the significant improvement in knowledge and total scores for both groups). However, there was no significant difference between the scores in post-test assessment with that of the 2 months post-training suggesting that the knowledge and skill retention in both arms were good.

Overall, participants in the gamification arm perceived the various game elements (points, leader boards, fun, teamwork, competition and immediate real-time feedback) and game mechanics (quiz-based format and level up progression via points, the added sense of enjoyability) favorably. They felt motivated to learn how to perform POCUS. This sense of motivation as well as the element of fun are postulated to be important impetus to fully engage the participants in their learning processes [16]. When the participants are deeply engaged or immersed in the activities they were participating in, they are said to be getting “into the flow”.

**Fig. 3:**
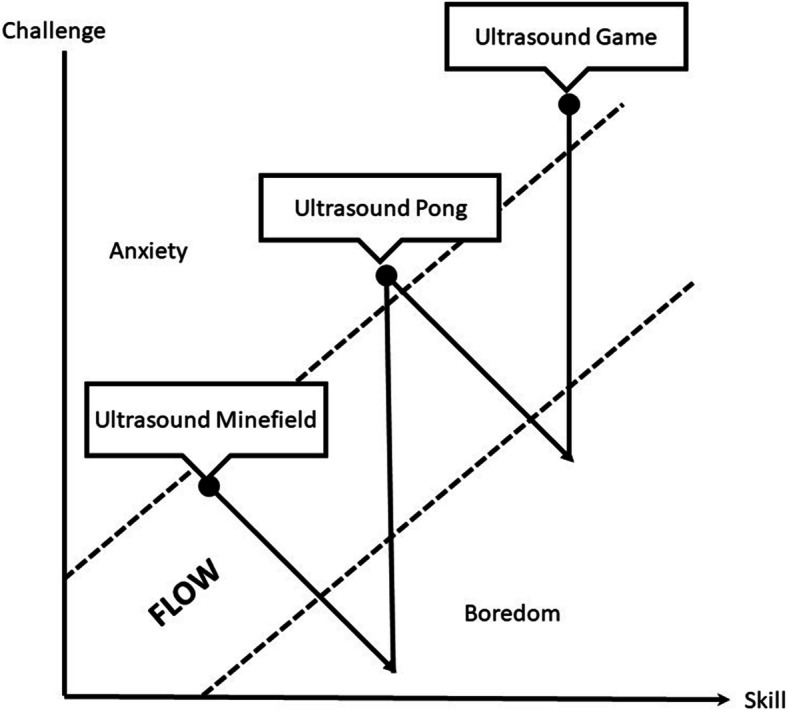
The concept of flow created by the various ultrasound games

Flow is a concept describing the experience people enjoys so much to the extent that their attention and focus are fully vested into the task at hand [17]. It is called “flow” because metaphorically, this is akin to someone being drifted effortlessly in the flow of water current without being aware of the passage of time or the fatigue feelings that he or she may have. In our study, this is suggested by the fact that all of our participants agreed that “… time passed by quickly during this course”. The state of flow can be harnessed when people were engaged in various types of games [18, 19]. According to Csikszentmihalyi et al. [17], three key conditions need to be fulfilled in order to achieve the engaging flow experience. First, there should be a clear set of goals to provide the trajectory and purposes of the activities. In this regard, the three ultrasound games have clear goals for the participants to achieve, i.e., acquiring the image and correctly calculate the number of water balloons hidden in a gelatin-filled container (in “ultrasound minefield”), demonstrating the image of the anatomy required as dictated in the ping-pong ball (in “ultrasound pong”) and competently performing POCUS assessment using the RUSH protocol in a simulated case scenario (in “ultrasound game”). Second, there should be a balance between the perceived challenges and perceived skills. When the perceived challenges and perceived skills are well matched, the attention is completely absorbed. When the perceived challenges begin to exceed the perceived skills, anxiety may set in. On the other hand, when the perceived skills exceed the perceived challenges, one may sink into boredom. In this regard, the game mechanics (the level up progression of the three ultrasound games tailored with increasing difficulty levels) sets to ensure that the participants have obtained the skills necessary in one level before progresses to the more challenging level (see Fig. 3 for an illustration of this concept). Third, just as what was pointed out by our participants, clear and immediate feedback should be given, regardless of whether it is positive performance feedback or negative performance feedback. In this regard, the game elements (such as the points, leader board and immediate comments from examiners) provide the immediate feedback to the participants.

Interestingly, majority of the CRUSH participants said that they enjoyed working with teammates and this sort of collaborative learning in turn, helped them to better acquaint themselves with their fellow colleagues. These 2 traits, i.e., teamwork and effective communication are essential in clinical management in the emergency department to deliver optimal health care. This suggests that although the CRUSH group did not seem to have an edge over the conventional group in terms of theoretical knowledge and practical skill acquisition (possibly due to the limited breadth of assessment), there may be other unmeasured benefits in the gamification approach such as the greater opportunity for collaborative learning.

As alluded by Bandura [20] in his social cognitive theory, optimal learning takes place in a social context, when participants learn and imitate from one another through social interactions, teamworking and communication. The World Health Organization (WHO) has accordingly also stressed on the importance in effective teamwork in the health care environment, as it is closely linked to reducing adverse events due to miscommunication and misunderstanding among teams caring for the patients [21].

This study, however, is subjected to several limitations. First, the sample size is relatively small. This decreases the power of the study and increases the risk of Type II errors. Furthermore, this study only included junior doctors from a single center. Inviting doctors of different competency level and from different centers in Malaysia to participate in such study would be more representative of a wider population and, therefore, bolster a stronger basis for consequent generalization of findings. Next, we could not exclude the possibility of the Hawthorne effect among the participants, as they were well aware that they were under evaluation in the research study to assess the effectiveness of gamification, thus may lead to a biased outcome. Although both arms have equal participants from almost similar working experience and backgrounds, there may be other confounding factors in play that may influence the study (such as the participant’s prior ultrasound knowledge and skills learned during their undergraduate studies). Moreover, the participants from both arms were combined in the lectures and pre-assessment skills training sessions. This might have a cross-contamination effect on the post-test performance through their interactions and discussions. Another confounding factor was prior to the 2 months post-training test, the participants could have read up more and attended other training workshops on ultrasound before the 2-month repeat assessment.

Future studies could look into the application of digital technology in streamlining game elements such as points, badges, live leader boards, avatars and virtual rewards and game mechanics like quizzes with immediate feedback and video clips as well as integrating social media use. Such studies could also be done in different populations, such as among specialists from different disciplines, nurses, paramedics and medical students to gauge its effectiveness in POCUS training. More comprehensive assessment (instead of limiting to a short theoretical assessment test and one OSCE case scenario) should be conducted to improve the validity of the effectiveness of gamification approach as compared to the conventional approach.

## Conclusion

This study shows that gamification approach could be an effective alternative to conventional approach in POCUS training, particular in the skill training. The incorporation of gamification into POCUS training can potentially be used as an engaging and enjoyable platform to deliver ultrasound training for junior doctors.

## Data Availability

The *.sav data that support the findings of this study are available in https://tinyurl.com/wytfjjb or from the corresponding author (KSC) upon reasonable request (should the link no longer works).

## Abbreviations

POCUS: Point-of-care ultrasound
RUSH: Rapid Ultrasound for Shock and Hypotension
CRUSH: Competition-based Rapid Ultrasound in Shock and Hypotension
ETD: Emergency and Trauma Department
SGH: Sarawak General Hospital
WINFOCUS: World Interactive Network Focused on Critical Ultrasound
OBA: One-best answer
OSCE: Objective structured clinical examination
XP: e**X**perience **P**oints
WHO: World Health Organization
